# Epidural Analgesia *With* or *Without* Oxytocin, but Not Oxytocin Alone, Administered During Birth Disturbs Infant Pre-feeding and Sucking Behaviors and Maternal Oxytocin Levels in Connection With a Breastfeed Two Days Later

**DOI:** 10.3389/fnins.2021.673184

**Published:** 2021-06-29

**Authors:** Yuki Takahashi, Kerstin Uvnäs-Moberg, Eva Nissen, Lena Lidfors, Anna-Berit Ransjö-Arvidson, Wibke Jonas

**Affiliations:** ^1^Department of Integrated Health Sciences, Nagoya University Graduate School of Medicine, Nagoya, Japan; ^2^Department of Animal Environment and Health, Swedish University of Agricultural Sciences, Skara, Sweden; ^3^Department of Women’s and Children’s Health, Karolinska Institutet, Stockholm, Sweden; ^4^Department of Global Public Health, Karolinska Institutet, Stockholm, Sweden

**Keywords:** epidural analgesia, newborn behavior, oxytocin, postpartum, rooting, breastfeeding

## Abstract

**Aims** This work aimed to study consequences of medical interventions in connection with birth on infant pre-feeding and feeding behaviors and on maternal oxytocin levels in connection with a breastfeed 2 days later.

**Materials and Methods** Mothers and their full-term newborns (*n* = 41) were videotaped during a breastfeed 2 days after birth. Duration and quality of rooting [Infant Breastfeeding Assessment Tool (IBFAT)] were assessed. Maternal blood samples were collected, oxytocin levels were analyzed, and mean oxytocin level and variance were calculated. Data on medical interventions during birth, number of breastfeedings, and infant weight loss since birth were recorded. Data were analyzed using logistic regression models.

**Results** The duration of infant rooting was significantly shorter when the mother had received epidural analgesia. The shorter the duration of infant rooting, the more often infants had breastfed and the greater was the infant weight loss since birth. Mothers with epidural analgesia *with* oxytocin had the lowest oxytocin mean levels in connection with a breastfeed. Oxytocin variance correlated positively with quality of rooting and correlated negatively with infant weight loss. In the control group alone, we found similar patterns of associations with oxytocin levels.

**Conclusion** Epidural analgesia and epidural analgesia *with* oxytocin infusion in connection with birth negatively influenced infant rooting behavior and maternal mean oxytocin levels, respectively. Oxytocin infusion alone was without effect. The data also suggest that infants who suck well stimulate oxytocin release more efficiently, as expressed by a high oxytocin variance, leading to a better stimulation of milk production and consequently to a reduced infant weight loss 2 days after birth.

## Introduction

The World Health Organization (WHO) (2017) recommends exclusive breastfeeding for the first 6 months of life and mother–infant skin-to-skin (SSC) contact immediately after birth, which facilitates breastfeeding (Dykes and Aryeetey, 2018).

When a healthy full-term newborn infant is placed in uninterrupted contact SSC with his/her mother immediately after normal and non-medicated birth, the newborn exhibits an organized pre-breastfeeding behavioral pattern. The infant starts to suck the breast at about 1 h of age and falls asleep at about 2 h of age (Widstrom et al., 1987).

Rooting is one of the most well-known and well-defined pre-feeding behaviors of the newborn. During rooting, the infant lifts his/her head and turns the head from side to side with concomitant opening of the mouth to prepare for sucking (Prechtl, 1958; Glodowski et al., 2019). Rooting behavior is stimulated when the infant cheek touches the maternal breast and increases in intensity when the infant wants to breastfeed. Activation of the rooting reflex is mediated by stimulation of sensory nerves in the skin of the infant cheek, but other stimuli such as hunger or odors can facilitate rooting behaviors (Varendi et al., 1997; Hym et al., 2020).

A previous study has demonstrated that infant rooting behaviors most likely predict the ability to express the infant’s need for food, i.e., to breastfeed. If the infant does not root or roots to a lesser extent, (breast)feeding ability is depressed. Thus, rooting is an internal feeding cue from the newborn, and for the mother, rooting behavior is a clear signal that the infant wants to breastfeed (Glodowski et al., 2019). Thus, if the rooting reflex is negatively affected by maternal medical interventions even on day 2 after birth, the infant might not latch on to the breast correctly, and the dyad may lose an opportunity to breastfeed.

Oxytocin is a peptide molecule which is produced in the supraoptic and paraventricular nuclei (SON and PVN) of the hypothalamus and is transported to the posterior pituitary and released into the circulation. The milk ejection reflex is stimulated when the infant starts to suck the breast (Lawrence and Lawrence, 2010). Sucking leads to an immediate and pulsatile release of oxytocin, which causes contractions of the myoepithelial cells that are located in the mammary glands and subsequent milk ejection occur. In addition, massage-like hand movements of the infant on the maternal breast is followed by increase of maternal oxytocin levels (Matthiesen et al., 2001).

In a recent study by our group, pre-feeding and sucking behaviors of the full-term newborn to unmedicated mothers were studied in connection with a breastfeed 2 days after birth (Takahashi et al., 2015). It was found that the duration of pre-feeding behaviors on day 2 after birth was much shorter than the duration of pre-feeding immediately after birth.

However, pre-feeding behaviors occurred in the same order as immediately after birth: rooting, hand-to-mouth movements, licking of the nipple, and hand-to-breast-to-mouth movements, where rooting was the most prominent behavior. Furthermore, low infant age and high weight loss since birth were related to a long duration of rooting, suggesting the existence of a physiological mechanism aimed at ascertaining adequate food intake (Takahashi et al., 2015).

The use of medical interventions has become more common during childbirth over the last decades. Epidural analgesia (EDA) is the most common pharmacological pain relief during labor in the Western world. For example, 62% of primiparous women in Sweden receive EDA at some stage of labor (National Board of Health and Welfare, 2019). Evidence has shown that medical interventions administered to the mother in connection with childbirth influence maternal and infant behavior and their interaction. For instance, opioids have been shown to delay the first breastfeeding after birth and to hamper the sucking behavior of the infant (Murray et al., 1981; Nissen et al., 1995). Epidural analgesia with bupivacaine administered to mothers negatively affected infants’ scores on the Brazelton Neonatal Behavioral Assessment Scale (BNBAS), where infants whose mothers had received EDA during birth showed poorer performance on motor clusters during the first month of life (Sepkoski et al., 1992). Exposure to regional anesthesia during birth has also been associated with impairment of infant motor functions of the mouth and limbs up to 6 weeks after birth (Baumgarder et al., 2003). Further, newborns to mothers who had been exposed to different types of analgesia such as bupivacaine via EDA, mepivacaine via pudendal block (PDB), and/or pethidine cry more after birth and are less likely to find the nipple and start sucking (Ransjo-Arvidson et al., 2001). Similar findings are described by several authors, who showed that sucking behaviors were reduced in infants whose mother had been exposed to EDA (Bell et al., 2010; Brimdyr et al., 2015; French et al., 2016). In addition, studies have shown that the first breastfeeding was delayed when the mother had had an EDA during birth (Wiklund et al., 2009; Romano, 2011).

Infusions of synthetic oxytocin are widely used as a medical intervention in maternity wards to induce or augment labor, and oxytocin is also recommended to prevent or treat postpartum bleeding [World Health Organization (WHO), 2018].

In a study by Brimdyr et al. (2019) on the effect of various medical interventions during birth (fentanyl, EDA, and infusion of oxytocin) on infant behavior during the first hour postpartum, it was found that fentanyl administered with an infusion of oxytocin was associated with altered newborn behavior including the first sucking after birth. However, in that latter study, no data on amounts of oxytocin given were presented, and in the statistical analyses, no corrections for use of concomitant EDA were made. In fact, a closer look at the data shows that the infants of mothers who had received infusions of oxytocin were more interactive than infants of control mothers; those infants crawled and familiarized for a longer period of time (Brimdyr et al., 2019). Another study suggested that intrapartum oxytocin infusion, administered to the mother during birth disturbs infant sucking behavior and breastfeeding duration, but the role of EDA was not discussed (Olza Fernández et al., 2012).

Hence, in previous studies, possible effects of EDA and oxytocin infusion have not been studied separately, and there are no studies showing that oxytocin given alone has a negative impact on infant behavior.

Additionally, medical interventions in connection with birth such as cesarean section and EDA together with oxytocin infusion negatively impact oxytocin levels 2 days after birth during breastfeeding (Nissen et al., 1996; Jonas et al., 2009).

In light of the findings above, our aims in the present study were threefold:

1) To explore whether intravenous (iv) infusion or intramuscular (im) injection of exogenous oxytocin or EDA *with* or *without* oxytocin administered to the mother during childbirth affected pre-feeding behaviors of the newborn 2 days after birth.
2) To explore if medical interventions in connection with birth and pre-feeding and sucking behaviors of the infant influence maternal oxytocin release in connection with a breastfeeding 2 days after birth.
3) To investigate whether infant weight loss and number of breastfeeding since birth influence the variables described above.

## Materials and Methods

### Participants and Setting

This study is part of a larger study conducted at a postnatal ward in Stockholm, Sweden (Jonas, 2009). Swedish-speaking, healthy, and non-smoking primiparous mothers with a body mass index below 30 were informed about the study. The infants were healthy and born at term and had an Apgar score of at least 8 at 1 min after birth. All newborns had been placed in skin-to-skin contact with their mother immediately after birth and had started breastfeeding within the first 2 h following birth. All mother–infant dyads had practiced rooming in and were not separated from their infants, and infants were exclusively breastfed during the stay at the maternity ward (Jonas, 2009). At this clinic, staff is instructed to interfere as little as possible in the care of the mother–infant dyad.

#### Study Sample

In the larger study, 72 mothers were included. However, in the present study, 41 mother–infant dyads participated (21 boys and 20 girls). The reasons for not being included were as follows: 16 mothers did not want to be video filmed, one infant had a fracture of the clavicle, and 14 films were of a quality that did not allow detailed assessment of behaviors of the newborn.

#### Medical Interventions: Group Allocation

All women had a spontaneous, vaginal birth and had skin-to-skin contact with their infants immediately after birth. All newborns had breastfed after birth. The mothers had been consecutively recruited to the study and were grouped based on which medical interventions they had received during labor. Thirteen mothers had received no medical intervention during labor, the control group (CTRL). Five mothers had received intravenous oxytocin infusion alone, the oxytocin group (OT iv). Eight women has received oxytocin as an intramuscular injection after birth, the OT im group (OT im). In total, 15 mothers had received EDA: five of those had received EDA alone (the EDA *without* OT iv group) and 10 mothers had received EDA *with* oxytocin infusion (the EDA *with* OT iv group).

#### Medical Interventions: Indications and Dose

If inertia was diagnosed during labor, oxytocin was administered intravenously (*n* = 15). The oxytocin infusion ampullae, containing 10 IU (16.6 μg) of oxytocin/ml (Syntocinon^®^, Novartis AB, Täby, Sweden) were diluted in 500 ml of Ringer’s acetate solution (Fresenius Kabi, Uppsala, Sweden), giving rise to a concentration of (20 mIU/ml or 33.3 ng/ml).

The median (Q_25_–Q_75_) amount of oxytocin given to mothers during birth was 1.86 (1.145–5.046) μg in the OT iv group and 4.16 (1.487–6.308) μg in the EDA with OT iv group.

According to local guidelines, all women received 10 IE oxytocin (Syntocinon) im after birth to prevent postpartum bleeding. During the study period, midwives were instructed not to administer oxytocin to the women fulfilling the inclusion criteria. However, some midwives did not comply with this instruction, and therefore, some mothers received 10 IE oxytocin im postpartum. These mothers constitute the OT im group.

When mothers were in need of pain relief, EDA was given. EDA consists of the local anesthetic bupivacaine (Marcain^®^, AstraZeneca, Södertälje, Sweden) in combination with the pain-relieving opiate sufentanil (Sufenta^®^, Janssen-Cilag, Sollentuna, Sweden). The median (Q_25_–Q_75_) duration (hours:minutes) of infusion of EDA was 2:59 (1:40–4:33) in the EDA without OT iv group and 4:23 (2:32–6:27) in the EDA with OT group. The median dose of bupivacaine was 17.5 (15.0–25.0) mg in the EDA without OT iv group and 17.5 (17.5–40.6) mg in the EDA with OT iv group, and the corresponding dose of sufentanil was 10.0 (10.0–15.0) and 10.0 (10.0–25.0) μg.

#### Video Recordings

Data were collected when the infants were between 24 and 48 h of age. Video recordings took place between 7 and 11 am. The mothers were instructed to call for the research midwives when they experienced by themselves that their baby wanted to breastfeed. No further detailed instructions were given. Video recordings of a breastfeed were conducted in the mothers’ rooms at the maternity ward. While video recording, conversation with the staff was kept to a minimum to allow undisturbed mother–infant interaction. During the video recording, the infants’ trunk and legs were covered with a light blanket. The video camera was directed to the upper part of the infant body and face and to the maternal chest and face during the recording. Data on the number of breastfeeding after birth were obtained through mothers’ self-reports, and clinical background data were collected from the birth records.

#### Coding Procedure

Video films were downloaded into the INTERACT software, version 9 (Mangold International, Germany 2011)^1^. One researcher (YT), blind to the mothers’ clinical and sociodemographic information, performed all assessments of infant breastfeeding sessions.

### Measures

#### Assessment of Pre-feeding Behaviors

Before sucking, newborns exhibited a number of pre-feeding behaviors (i.e., rooting, hand-to-mouth movements, hand-to-breast-to-mouth movements, licking of the nipple, and pressing on mother’s breast). The duration of the first sucking period (i.e., the first intense period between start of sucking and first latch-off) was assessed. These behaviors are described in Table 1.

**TABLE 1:** Pre-feeding behaviors and their definitions.

**Pre-feeding behaviors**	**Definitions**
Rooting movements	The infant moves/lifts his/her head, turns it from side to side without having the nipple inside the mouth
Hand-to-mouth movements	The infant finger(s) is/are inside his/her mouth or touch(es) his/her lips
Hand-to-breast-to-mouth movements	The infant hand touches the breast/areola and brings his/her hand back to its own mouth
Licking of the nipple	The infant lips and/or the tongue are in contact with the nipple
Pressing on maternal breast	The infant uses the palm of his/her hand and/or his/her fist to press on the mother’s breast, just before onset of sucking

#### Infant Breastfeeding Assessment Tool (IBFAT)

The Infant Breastfeeding Assessment Tool (IBFAT), a validated and well-established study protocol to assess infant feeding competence (Matthews, 1988), was used for assessing the quality of pre-feeding and breastfeeding behaviors. The IBFAT consists of four items and assesses readiness to feed or arousability, rooting, latching on, and sucking. The score for each single item ranged from 0 to 3, allowing a maximum of 12. Breastfeeding was considered effective when the total IBFAT score was 10–12, moderately effective when the score was 7–9, and less effective when the score was 0–6. The description of the subscales of the IBFAT protocol and the scoring are presented in Table 2.

**TABLE 2:** Infant breastfeeding assessment tool (IBFAT)*.

**Behavioral domains**	**Scores**			
	**3**	**2**	**1**	**0**
Readiness to feed	The baby started to feed readily without effort	Cannot be aroused	Needed more vigorous stimulation to rouse and start to feed	Could not be aroused
Rooting	Roots effectively at once	Needs some coaxing, prompting, or encouragement	Rooted poorly, even with coaxing	Did not try to root
Latch on	Feeds immediately	Takes 3–10 min to start	Takes over 10 min to start	Did not feed
Sucking pattern	Sucked well on both breasts	Sucks on and off, but needed encouragement	Weak sucks, sucks on and off for short periods	Did not suck

According to the Infant Breastfeeding Assessment Tool (IBFAT), breastfeeding was considered as effective at scores 10–12, moderately effective at scores 7–9, and less effective at scores 0–6.*Developing an instrument to assess infant breastfeeding behavior in the early neonatal period (Matthews, 1988).

Inter-observer reliability was tested for the two instruments by two independent observers (YT and ABRA) who assessed 10% of the video films. Interrater reliability was high across the assessments (82–96% for the pre-feeding variables and 100% for the IBFAT items).

#### Measurement of Oxytocin Levels

Blood samples were collected from an intravenous cannula inserted into the cubital vein in connection with the breastfeeding experiment performed 24–48 h after birth. The first blood sample was drawn immediately after the newborn started to suck the breast and was considered as the basal sample (sample 0). Then 15 samples were taken with 30-s intervals during the first 7.5 min, and thereafter, four samples were collected at 10, 20, 30, and 60 min after the start of sucking (20 samples in total). The samples were analyzed for oxytocin using the Correlate-EIA^TM^ Oxytocin Enzyme Immunoassay Kit according to the manufacturer’s instructions (sensitivity, 4.68 pg/ml; precision, 10.2%) (Assay Designs, Inc. Ann Arbor, MI, United States). Plasma samples were diluted five times in the assay buffer before analysis of oxytocin. Standards and controls were included on each plate as recommended by the manufacturers. The washing procedure was performed using an Anthos Fluido microplate washer (Anthos Labtec Instruments GmbH, Salzburg, Austria), and the absorbance was read using a Multiskan EX microplate photometer (Thermo Electron Corp., Vantaa, Finland). The color development of the samples was read at 405 nm. Background correction was measured at 580 nm. Ascent software (version 6 for iEMS Reader MF and Multiskan, Thermo Electron Corp) was used for creation of standard curves, curve fitting, and calculation of concentrations.

In the present study, we used ELISA as a method of analysis, without previous extraction of the samples. Much higher basal levels are detected using ELISA without previous extraction of the samples compared with using RIA. Although there is a difference in the size of basal oxytocin levels, the pattern of oxytocin release did not differ from the ones observed in studies in which RIA was used (Nissen et al., 1995). Using both methods, about four to five oxytocin peaks occurred during the first 7.5 min of sucking. This methodological problem has been discussed in detail in previous articles (Jonas et al., 2009).

### Data Analysis

Descriptive statistics were used to summarize the maternal and infant background characteristics and breastfeeding variables including pre-feeding behaviors and IBFAT subscale and total scores. The infant behavior was observed during 10-s periods for the last 60 s before sucking. The sum of the observations for each period was used for further calculation.

Data in general were described by medians and interquartile distances (Q_25_–Q_75_) or as frequencies and percentages (%). Mean oxytocin levels (20 samples) were presented as means (m) and standard errors (±SE). Oxytocin variance was assessed as the square of the standard deviation and was calculated for each mother (16 samples). The oxytocin variance is presented as means (±SE) and was used for further analysis of the oxytocin pattern.

Statistical testing was performed with SAS (Statistical Analysis System Inc., Cary, NC, United States, version 9.4). The distribution of data was assessed before analysis. Duration of rooting had a Poisson distribution. Mean oxytocin levels (0–60 min) had a skewed distribution and was therefore log-transformed. Oxytocin variance had a normal distribution.

We performed logistic regression analyses using a generalized linear model (Proc genmod) for all variables except IBFAT rooting, where an ordinal logistic regression (Proc logistic) was used. Parameters that were tested are presented in the section “Results.” A probability value of *p* ≤ 0.05 was considered significant.

### Ethical Considerations

The Ethics Committee at Karolinska Institutet, Stockholm, Sweden, had approved the study. All women provided written, informed consent.

## Results

### Clinical Characteristics

Maternal and infant background characteristics and observational data such as the number of breastfeeding and infant weight loss since birth and infant age at the observed breastfeeding day 2 are given in Table 3. There were no statistical differences in background characteristics between the groups (data not shown).

**TABLE 3:** Clinical characteristics of all mothers and their infants and as a function of the medical interventions the mothers had received during childbirth (medians, interquartile ranges Q_25_–Q_75_).

	**All (*n* = 41)**			**CTRL (*n* = 13)**			**OT iv (*n* = 5)**		
	**Median**	**Q_25_**	**Q_75_**	**Median**	**Q_25_**	**Q_75_**	**Median**	**Q_25_**	**Q_75_**
Maternal age (years)	30	27.5	32.5	31	27	33	32.0	28.0	33.0
Duration of labor (hours:minutes)	11:19	7:01	14:06	8:48	7:01	14:00	12:42	11:37	18:52
Bleeding (ml)	400	335	540	380	300	500	375.0	350.0	400.0
Use of N2O (yes/no)		30/11			5/8			5/0	
Acupuncture (yes/no)		12/29			3/10			1/4	
Number of boys/girls		20/21			7/6			2/3	
Birth weight (g)	3667.5	3231.3	4010	3500	3216.3	3811.3	4030	3055	4152.5
Infant weight loss (%) from birth until the observed breastfeed	−3.4	−5.3	−2.7	−3.3	−4	−1.6	−5.3	−6.2	−3.9
Infant age at observed breastfeed (hours)	36	30	40	30	25	36	36	34.5	45
Number of breastfeeds since birth	7	5	9	7	4	10	6	5	11.5
	**OT im (*n* = 8)**			**EDA *without* OT (*n* = 5)**			**EDA *with* OT (*n* = 10)**		
	**Median**	**Q_25_**	**Q_75_**	**Median**	**Q_25_**	**Q_75_**	**Median**	**Q_25_**	**Q_75_**
Maternal age (years)	27.5	25.5	29	31	26.0	31.5	31.0	29.8	34.3
Duration of labor (hours:minutes)	9:04	5:37	11:54	14:06	4:37	28:46	10:56	8:05	13:34
Bleeding (ml)	460	325	787.5	500	275	655	440	387.5	797.5
Use of N2O (yes/no)		7/1			4/1			9/1	
Acupuncture (yes/no)		1/7			2/3			5/5	
Number of boys/girls		4/4			2/3			6/4	
Birth weight (g)	3980	3700	4236.3	3215	3177.5	4065	3685	3332.5	3776.8
Infant weight loss (%) from birth until observed breastfeed	−3.6	−6.7	−2.2	−4	−7.9	−0.7	−3.3	−5.1	−3.1
Infant age at observed breastfeed (hours)	32.5	22	41.5	33	30.5	47	38	32.5	43.3
Number of breastfeeds since birth	5.5	4	8.8	6	4	8.5	7.5	5	10.5

### Observational Data

#### Duration of Pre-feeding Behaviors and of the First Sucking Period

The total duration of pre-feeding behaviors and the first sucking period was recorded. In addition, the duration of individual subcomponents of the pre-feeding behaviors (rooting, hand-to-mouth movement, hand-to-breast-to-mouth movement, licking of the nipple, and pressing on mother’s breast), during the last 60 s before the start of sucking, was recorded. Values are presented for all four intervention groups and the control group (OT iv, OT im, EDA *without* OT, EDA *with* OT, and CTRL) in Table 4.

**TABLE 4:** Duration (seconds) of pre-feeding behaviors during the last minute before the onset of breastfeeding and the duration of all pre-feeding behaviors (minutes) and of the first sucking period (minutes) (median, interquartile ranges Q_25_-Q_75_), (*n* = 41) in all infants and as a function of the medical interventions their mothers had received during childbirth.

	**All (*n* = 41)**			**CTRL (*n* = 13)**			**OT iv (*n* = 5)**		
**Duration of specific pre- and feeding behaviors (seconds and minutes)**	**Median**	**Q_25_**	**Q_75_**	**Median**	**Q_25_**	**Q_75_**	**Median**	**Q_25_**	**Q_75_**
Rooting movements	17.4	5	29.3	21.1	12.7	36.3	21	8.9	39.3
Hand-to-mouth movements	0	0	4.5	0	0	2.9	0	0	17.6
Hand-to-breast-to-mouth movements	0	0	0.5	0	0	0	0	0	0
Licking of the nipple	2.2	0	15.6	0	0	10.1	8.7	2.4	14.6
Pressing on maternal breast	0	0	0.4	0	0	0.5	0	0	0
All pre-feeding behaviors (minutes)	2.4	0.7	8	1.8	0.5	7	2.6	1.4	9.4
Duration of first sucking period (minutes)	10	4.2	17.3	8.9	5.4	20	13.5	5.4	19.2
	**OT im (*n* = 8)**			**EDA *without OT* (*n* = 5)**			**EDA *with OT* (*n* = 9)**		
**Duration of specific pre- and feeding behaviors (seconds and minutes)**	**Median**	**Q_25_**	**Q_75_**	**Median**	**Q_25_**	**Q_75_**	**Median**	**Q_25_**	**Q_75_**
Rooting movements	17.5	4.2	26.6	10.1	5.7	29.4	9.5	0	27.3
Hand-to-mouth movements	1.7	0	12.2	0	0	0	0	0	4.7
Hand-to-breast-to-mouth movements	0.5	0	1.6	0	0	0	0	0	2
Licking of the nipple	8.3	0.6	25.1	14	6.1	29.1	0	0	4.2
Pressing on maternal breast	0.0	0	0.4	0	0	3.1	0	0	1.4
All pre-feeding behaviors (minutes)	1.0	0.5	6	3	1.5	8.5	4.9	0.4	13
Duration of first sucking period (minutes)	8.6	6.9	13.7	10	1.8	11.3	11.7	0	23.8

#### IBFAT Scores

The quality of infant pre-feeding behaviors and sucking as assessed by the IBFAT (readiness to feed, rooting, latching on, and sucking) is presented as medians and (Q_25_–Q_75_) in Table 5. Median total IBFAT scores varied between 8 and 10.5 and were the lowest in the EDA *without* OT group. The median of the subscale rooting was the lowest in the EDA without OT iv and in the EDA with OT iv groups (Table 5).

**TABLE 5:** IBFAT subscale scores and total scores of all infants and as a function of the medical interventions the mothers had received during childbirth.

	**All (*n* = 41)**			**CTRL (*n* = 13)**			**OT iv (*n* = 5)**		
**IBFAT subscale scores and total scores**	**Median**	**Q_25_**	**Q_75_**	**Median**	**Q_25_**	**Q_75_**	**Median**	**Q_25_**	**Q_75_**
Readiness to feed	3	2	3	3	2	3	3	2	3
Rooting	2	1	2	2	1.5	2.5	2	1	2
Latch-on	3	2	3	3	2	3	3	1.5	3
Sucking pattern	3	1	3	3	2	3	2	1.5	3
Total	10	7	11	10	8	11	10	7	10
	**OT im (*n* = 8)**			**EDA *without OT* (*n* = 5)**			**EDA *with OT* (*n* = 10)**		
**IBFAT subscale scores and total scores**	**Median**	**Q_25_**	**Q_75_**	**Median**	**Q_25_**	**Q_75_**	**Median**	**Q_25_**	**Q_75_**
Readiness to feed	3	2.3	3	2	1.5	3	2.5	0.8	3
Rooting	2	1.3	2.8	1	1	1.5	1	0	2
Latch-on	3	2.3	3	2	2	2.5	2	0	3
Sucking pattern	3	1.3	3	2	1	2.5	3	0	3
Total	10.5	8.5	11	8	6	8.5	9	0.8	11

### Factors That Influence the Duration of Infant Rooting and IBFAT Scores

In the present study, we wanted to explore in detail how medical interventions in connection with birth influenced pre-feeding behaviors before the start of breastfeeding 2 days after birth. Rooting behaviors were chosen for further analysis, since they occurred in most mother–infant dyads and as the duration of rooting seemed to vary between the groups receiving different medical interventions. We also explored the role of percentage of infant weight loss and the number of breastfeeding since birth on the duration and quality of rooting.

#### Factors That Influence the Duration of Rooting

The association between the duration of rooting and the different medical interventions the mothers had received during birth, the number of breastfeeding (≤ 7 and ≥ 8), and the percentage of weight loss in the infants (covariate) from birth until the observed breastfeeding 2 days after birth was investigated using a generalized linear model.

**FIGURE 1:**
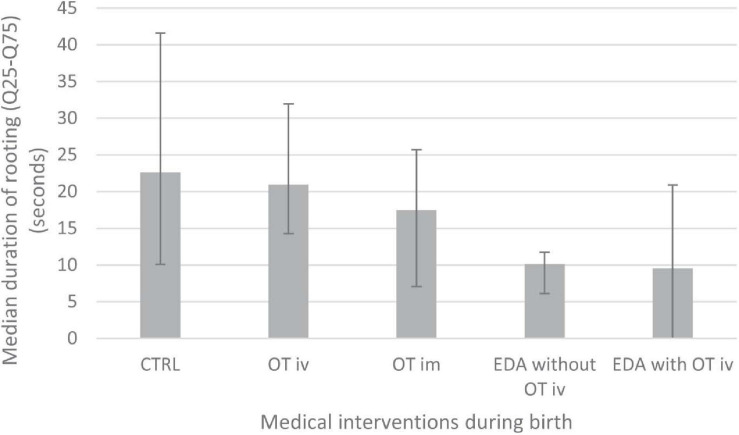
Shows the duration of rooting (seconds) during the last 60 s before start of the observed breastfeed 2 days after birth in the different intervention groups (CTRL, OT iv, OT im, EDA *without* OT iv or EDA *with* OT iv). Medians and interquartile ranges (Q_25_-Q_75_) are shown. Logistic regression analysis using a generalized linear model (PROC GENMOD) with a *post hoc* test with pair wise comparisons between groups were performed showed differences in rooting duration between the following groups: CTRL vs. OT iv (*p* < 0.05) and CTRL vs. OT im, EDA *without* OT iv and EDA *with* OT iv (*p* < 0.0001); OT iv vs. EDA *without* OT iv (*p* < 0.0001); OT iv vs. EDA *with* OT iv (*p* < 0.05), OT im vs. EDA *without* OT iv (*p* < 0.01) and EDA *without* OT iv vs. EDA *with* OT iv (*p* < 0.05).

There was a significant effect of medical interventions on the duration of rooting before the start of sucking (*p* < 0.0001, χ^2^ = 58.91, DF = 4). *Post hoc* tests revealed that infants of mothers who were not exposed to any medical intervention (CTRL) showed a significantly longer duration of rooting than the infants in the four intervention groups (OT iv, OT im, and EDA *with* and *without* OT iv) (Figure 1). Further, the duration of rooting was also significantly longer in infants whose mothers had received OT iv compared to those whose mothers had received EDA *with* or *without* OT iv in connection with birth. In addition, infants of mothers who had received OT im had significantly longer rooting duration compared to those mothers who had received EDA *without* OT iv. The duration of rooting in infants of mothers who had received EDA *without* OT iv differed significantly from infants of mothers who had received EDA *with* OT iv (Figure 1). The shortest duration of rooting was observed in those infants whose mothers had received EDA alone.

In addition, there was a significant effect of number of breastfeedings on the duration of rooting before the start of sucking (*p* < 0.0001, χ^2^ = 67.11); infants who had had seven or less breastfeeding sessions before the observed breastfeeding (*n* = 23) showed a significantly longer duration of rooting [median (Q_25_–Q_75_): 24.04 (6.12–46.76) seconds] compared to infants with eight or more breastfeeding sessions (*n* = 16) [median (Q_25_–Q_75_): 13.02 (0.64, 18.24) seconds]. In other words, the more breastfeeding sessions the infants had experienced before the observed breastfeeding 2 days after birth, the shorter the duration of rooting.

There was also a significant effect of percentage of weight loss since birth (*p* < 0.0001, χ^2^ = 25.91) on the duration of rooting before the start of sucking. The maximum likelihood estimate for percentage of weight loss was negative (−0.1377, *SE* = 0.0270), meaning that the higher the percentage of weight loss in the infants, the shorter the duration of rooting.

#### Factors That Influence IBFAT Rooting Scores

The association between the quality of rooting (as assessed by the IBFAT) and the different medical interventions the mothers had received during birth, the number of breastfeeding (≤7 and ≥8), and the percentage of weight loss in the infants (covariate) from birth until the observed breastfeeding 2 days after birth was investigated using ordinal logistic regression.

There was no significant association between the scores of IBFAT rooting and the medical interventions the mothers had received (*p* = 0.106, χ^2^ = 7.64) or the number of breastfeeding after birth (*p* = 0.61, χ^2^ = 0.26).

A *post hoc* test comparing the control group with the different medical intervention groups was performed. It was found that the EDA *with* OT iv group was significantly different from the control group (*p* = 0.02, χ^2^ = 5.22). Odds ratio (OR) estimates showed that infants born to mothers who had received EDA *with* OT iv had a lower IBFAT rooting scores than controls (EDA *with* OT iv, OR = 0.131; CI = 0.023–0.749).

#### Conclusion on Which Factors Affect Pre-feeding Rooting

In conclusion, these two analyses above show that the medical interventions received by the mother in connection with birth influenced the duration of the infant’s rooting behavior preceding sucking (and the quality of rooting as assessed by the IBFAT score). The EDA *without* OT iv group showed the shortest duration of rooting. (The EDA *with* OT iv group had the lowest rooting scores on the quality assessment tool IBFAT). By contrast, administration of oxytocin either iv or im did not influence the infant’s rooting behavior.

In addition, the number of breastfeedings before the observation, performed 2 days after birth, influenced the duration of rooting behavior; i.e., the more breastfeeding after birth, the shorter the duration of rooting. Additionally, the higher the weight loss in the infants since birth, the shorter the duration of rooting.

### Factors That Influence Oxytocin Levels During Breastfeeding

Infants’ rooting behavior is an essential component of infants’ pre-feeding behaviors and thereby associated with initiation of breastfeeding, which is known to trigger maternal oxytocin release. It was therefore of interest to investigate if the duration or quality of infant’s pre-feeding behaviors is linked to maternal oxytocin levels in connection with breastfeeding. It was also of interest to investigate a possible relationship between the use of medical interventions during birth, such as administration of exogenous oxytocin or of EDA, as well as the number of breastfeedings and the weight loss of the infant since birth.

We used two types of oxytocin measures that express different characteristics of the oxytocin profile. Oxytocin mean levels (0–60 min) to a large extent reflect the maternal individual characteristics, whereas oxytocin variance (0–7.5 min) to a large extent reflects infant sucking activity and capability.

Therefore, in the following analyses, we explored whether medical interventions administered to the mother during birth, duration (covariate) or quality of infant rooting, duration of sucking (covariate), the number of breastfeedings (≤ 7 and ≥ 8), and percentage of weight loss (covariate) were associated with maternal mean oxytocin levels or oxytocin variance during the observed breastfeeding 2 days after birth. We used a generalized linear model.

#### Maternal Mean Oxytocin Levels (0–60 min) During Breastfeeding

**FIGURE 2:**
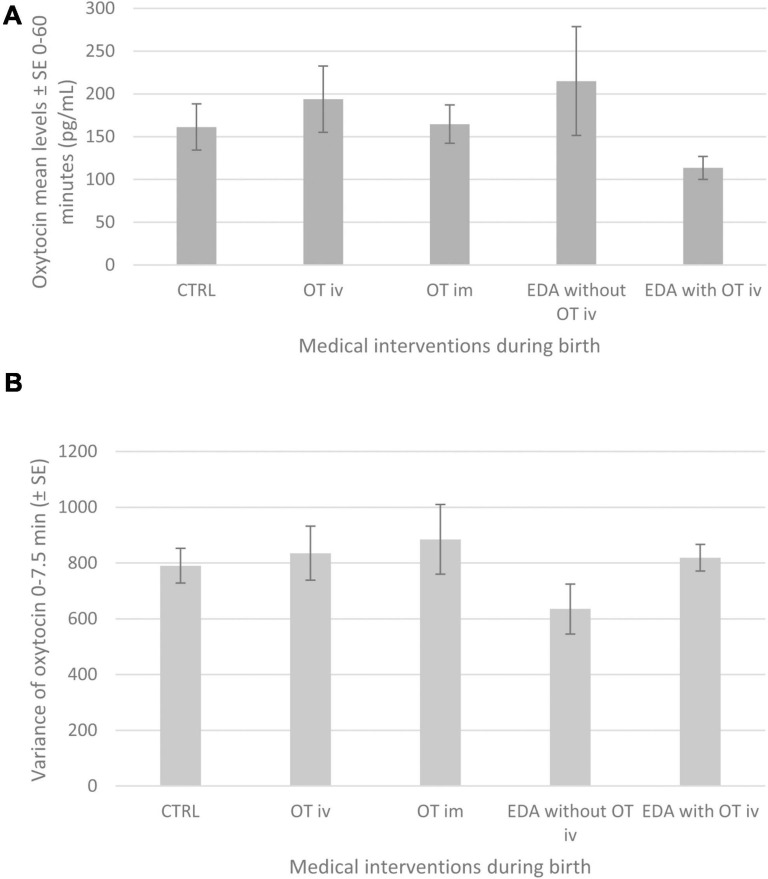
**(A)** Shows maternal mean (±SE) plasma levels of oxytocin (pg/mL) from 0 to 60 min during the observed breastfeed in mothers 2 days after birth in the different intervention groups: CTRL, OT iv, OT im, EDA w*ithout* OT iv or EDA *with* OT iv. Logistic regression analysis using a generalized linear model (PROC GENMOD) with a *post hoc* test where pair wise comparisons between groups were performed: CTRL vs. EDA *with* OT iv (*p* < 0.05), OT iv vs. EDA *with* OT iv (*p* < 0.05), OT im vs. EDA *with* OT iv (*p* < 0.05) and EDA *without* OT iv vs. EDA *with* OT iv (*P* < 0.01). **(B)** The figure shows maternal mean variance (±SE) of oxytocin (pg/mL) from 0 to 7.5 min during the observed breastfeed in mothers 2 days after birth in the different intervention groups: Logistic regression analysis using a generalized linear model (PROC GENMOD) did not find any significant effect of the medical interventions and therefore these data are only shown descriptively.

The statistical analysis showed that maternal mean levels of oxytocin in connection with a breastfeeding 2 days after birth were significantly associated with the medical interventions the mothers had received during birth (*p* = 0.02) (Table 6). The mean oxytocin levels in mothers who had received EDA *with* OT iv during birth were lower than in mothers who had received any of the following medical interventions during birth—OT iv (*p* < 0.05), OT im (*p* < 0.05), and EDA *without* OT iv (*p* < 0.001)—or in control mothers, CTRL (*p* < 0.05). Mean oxytocin levels (0–60 min) in the different groups in connection with a breastfeeding 2 days after birth are shown in Figure 2A.

**TABLE 6:** The relationship between **(A)** maternal mean oxytocin levels 0–60 min (log transformed) and **(B)** oxytocin variance 0–7.5 min of the observed breastfeed 2 days after birth and the following predictors; *medical interventions during labor, number of breastfeeds since birth* (<7 or >8), *IBFAT Rooting score, % of weight* loss in infants since birth (covariate) and *duration of the first sucking period* (covariate).

		**(A) Mean oxytocin 0-60 min**		**(B) Variance of oxytocin 0-7.5 min**	
**Predictors**	**DF**	**χ^2^**	***p*-value**	**χ^2^**	***p*-value**
Medical interventions	4	11.61	**0.02**	3.76	0.44
Number of breastfeeds since birth	1	2.05	0.15	1.64	0.20
IBFAT rooting score	3	6.67	0.08	3.6	0.31
% of weight loss	1	5.79	0.07	5.11	**0.02**
Duration of sucking period	1	4.49	**0.03**	0.32	0.57

*Bold values indicate significant differences (*p* < 0.05).*

Oxytocin levels in connection with the observed breastfeeding 2 days after birth were significantly associated with the duration of sucking (*p* = 0.03) (Table 6). This association was positive (maximum likelihood estimate = 0.011, *SE* = 0.0051), indicating that the longer the duration of sucking, the higher the mean oxytocin levels.

There were no significant associations between mean oxytocin levels and the number of breastfeeding since birth, IBFAT rooting score, and percentage of infant weight loss (Table 6).

#### Variance of Maternal Oxytocin Levels (0–7.5 min) During Breastfeeding

There was a significant association between variance of maternal oxytocin levels and percentage of weight loss in the infants since birth (*p* = 0.02). The association was negative (maximum likelihood estimate = −68.26, *SE* = 30.18), indicating the higher the percentage of weight loss in the infants, the lower the variance of maternal oxytocin levels (0–7.5 min).

There was no significant association between the mean variance of maternal oxytocin levels and exposure to medical interventions during birth, number of breastfeeding since birth, IBFAT rooting score, and duration of sucking period (Table 6). Figure 2B shows means ± SE of variance of maternal oxytocin for the different medical interventions.

### Factors That Influence Oxytocin Levels and Variance in the Control Group

Medical interventions administered to the mother in connection with birth influenced maternal oxytocin mean levels during a breastfeeding 2 days later. We therefore wanted to explore the control group by itself, since this group is devoid of such influences. The same analysis as used for the entire group and as described in sections “Maternal Mean Oxytocin Levels (0–60 min) During Breastfeeding” and “Variance of Maternal Oxytocin Levels (0–7.5 min) During Breastfeeding” above was performed in the control group alone.

#### Maternal Mean Oxytocin Levels (0–60 min) During Breastfeeding

The number of breastfeeding since birth in control mothers was significantly associated with mean oxytocin levels (*p* = 0.0001) (Table 7). Mean oxytocin levels were significantly higher in mothers when the infants had seven or less breastfeedings since birth (means ± SE: 179.9 ± 28.04 pg/ml) than when they had eight or more breastfeedings (means ± SE: 128.9 ± 58.71 pg/ml). Thus, a low number of breastfeedings was associated with higher mean oxytocin levels.

**TABLE 7:** The relationship between **(A)** maternal mean oxytocin levels 0–60 min (log transformed) and **(B)** oxytocin variance 0–7.5 min of the observed breastfeed 2 days after birth in the control group (*n* = 12) alone and the following predictors; *number of breastfeeds since birth* (<*7* or >8), IBFAT Rooting score, % of weight loss in infants since birth (covariate), and duration of the first sucking period (covariate).

		**(A) Mean oxytocin 0–60 min**		**(B) Variance of oxytocin 0–7.5 min**	
**Predictors**	**DF**	**χ^2^**	***p*-value**	**χ^2^**	***p*-value**
Number of breastfeeds	1	26.28	**0.0001**	0.67	0.41
IBFAT rooting score	2	4.91	0.09	3.43	0.18
% of weight loss	1	22.81	**0.0001**	6.35	**0.012**
Duration of first sucking period	1	2.26	0.13	0.74	0.39

*Bold values indicate significant differences (*p* < 0.05).*

There was also a significant association between percentage of infant weight loss since birth and mean oxytocin (*p* = 0.0001) (Table 7). The association was negative (maximum likelihood estimate = −0.50, *SE* = 0.106), meaning that the higher the percentage of infant weight loss, the lower the oxytocin levels.

There were no significant associations between mean oxytocin levels and IBFAT rooting score and duration of sucking (Table 7).

#### Variance of Maternal Oxytocin Levels (0–7.5 min) During Breastfeeding

The variance of oxytocin in control mothers was significantly associated with the percentage of weight loss since birth in the infants (*p* = 0.012) (Table 7). The association was negative (maximum likelihood estimate = -101.17, *SE* = 40.15), meaning that the higher the percentage of weight loss, the lower the variance of oxytocin.

There was no significant association between variance of oxytocin levels and number of breastfeeding, IBFAT rooting score, and duration of sucking (Table 7).

## Discussion

### Summary

In the present study, the duration of some pre-feeding behaviors during the last minute before the start of sucking and IBFAT scores, a qualitative measure of infant breastfeeding performance, were assessed in connection with a breastfeeding 2 days after birth.

Regression analyses revealed that duration and quality of rooting were negatively influenced by both EDA alone and by EDA together *with* infusion of oxytocin administered to the mothers in connection with labor and birth. By contrast, no effect on rooting was observed by administration of oxytocin iv or im. In addition, the more often the infants had breastfed and the greater their weight loss since birth, the shorter the duration of infant rooting.

We also assessed maternal oxytocin levels (mean and variance were calculated) in connection with the same breastfeeding.

Regression analysis showed that there was a significant influence by medical interventions during birth on mean oxytocin levels in connection with breastfeeding. Mothers who had received EDA *with* oxytocin infusion had the lowest mean oxytocin levels. The duration of sucking was positively associated with mean oxytocin levels. Medical interventions did not influence oxytocin variance, but the variance was lower the higher the weight loss of the infants.

When similar analyses regarding oxytocin levels were performed in the control mothers alone, i.e., in those mothers who had not received any medical intervention during birth, some new associations were found; low mean oxytocin levels were associated with a low number of breastfeedings since birth and were also associated with greater infant weight loss since birth. Oxytocin variance was lower the greater the infant weight loss since birth.

### Pre-feeding Behaviors of the Newborn

#### Effect of Medical Interventions in Connection With Birth

Infants of mothers who had received EDA *without* oxytocin administration displayed significantly less rooting behavior during the 60 s preceding the breastfeeding session than those who had not received EDA or EDA *with* oxytocin. In addition, the quality of rooting was significantly lower in the group of infants whose mothers had received EDA *with* oxytocin.

We observed a shortened period of rooting on day 2 after birth, indicating that rooting had been depressed since birth. The fact that the rooting period was shorter may be one of the reasons for the lower weight gain observed in infants whose mothers had received EDA in connection with birth.

The finding of an impaired infant rooting behavior in the present study is in accordance with data from a previous study, in which infants to mothers who had received mepivacaine or bupivacaine, e.g., in connection with administration of EDA, displayed a deranged interactive behavior with their mother after birth (Ransjo-Arvidson et al., 2001). This effect could be explained by the fact that local anesthetics easily pass the placental barrier and may thus have direct effects on the newborn’s behavior. It is however more difficult to understand how these compounds would affect the newborn’s behavior 2 days later, since it is not likely that mepivacaine or bupivacaine are still present in the circulation of the newborn. It is, however, possible that drugs, like the ones used in the present study, will exert long-term effects in connection with birth by imprinting or induction of epigenetic mechanisms. Indeed, long-term effects have been shown to occur in response to interventions such as skin-to-skin contact during the period surrounding birth (Bystrova et al., 2009; Moore et al., 2016; Bigelow and Power, 2020; Uvnas-Moberg et al., 2020).

Alternatively, the effects observed on the rooting behavior of the newborn are indirect and operate through a changed physiology or through the behavior of the mother. This assumption is supported by the fact that skin temperature did not increase in the infants of the present study in connection with breastfeeding (Jonas et al., 2007), which usually is the case (Bystrova et al., 2003, 2007). It is therefore possible that mothers who had received EDA did not emit as strong physiological signals as warmth or pheromone cues to the baby as those mothers who had not received EDA.

It has been suggested that oxytocin administered to the mothers in connection with birth would influence the newborn’s behavior in a negative way and that administration of oxytocin would negatively impact the development of the infants’ sucking behavior (Olza Fernández et al., 2012; Brimdyr et al., 2019). These studies however do not convincingly show that oxytocin negatively influences the behavior of the newborn. When the study by Brimdyr et al. (2019) is analyzed in detail, the newborns to mothers who had been exposed to oxytocin were in fact more interactive and had a longer crawling and familiarization phase than controls (Brimdyr et al., 2019). Another study suggested that infant motor development was disturbed in response to oxytocin infusion during labor. However, the impact of concomitant administration of EDA was not analyzed, and it is therefore likely that it was the EDA component that contributed to this negative effect (Olza Fernández et al., 2012). In the present study, we did not find any support for negative effects of oxytocin given as intravenous infusion on infant behavior.

#### Link Between Rooting Behavior and Infant Weight Loss Since Birth

We also found a negative correlation between duration of rooting and weight loss of the infant since birth, i.e., the more weight the infant had lost, the less rooting they performed. These results are in contrast to previous findings based on this study (Takahashi et al., 2015). The study by Takahashi et al. (2015) included only mothers who had *not* received any medical interventions in connection with birth, and rooting duration was longer the younger the infants were and the more weight they had lost 2 days after birth. We interpreted this finding as an expression of a physiological adaptation to increase food intake and thereby weight gain, which would be of critical importance not only in young infants but also in those who have been exposed to a substantial loss of weight. As mentioned above, the findings of the present study show an inverse relationship between rooting and weight loss after birth. The finding in this study suggests that the physiological adaptation, allowing compensation for high weight loss, was lost or disturbed when studied in the whole group of mothers. It is likely that the pronounced decrease of the duration of rooting caused by EDA plays a central role in the inversion of the relationship between duration of rooting and size of infant weight loss since birth which was found in the whole group.

### Maternal Oxytocin Levels

#### Oxytocin Mean Levels Versus Oxytocin Variance

Both mean oxytocin levels and oxytocin variance were used when analyzing the data of the present study, since it can be assumed that these two measures depict partly different aspects of the oxytocin release pattern in connection with sucking. Oxytocin levels rise almost immediately in response to sucking/breastfeeding and exhibit a peak-shaped response during 20 min, and then oxytocin levels return to basal levels (Nissen et al., 1996; Jonas et al., 2009). Mean oxytocin levels represent an average of oxytocin levels from 0 to 60 min. Oxytocin variance on the other hand is based on the values obtained during the first 7.5 min. As the peaks of oxytocin occur immediately in response to sucking, we assumed that oxytocin variance may to a larger extent reflect infant sucking activity than oxytocin mean levels, which is based on the entire 60-min-long breastfeeding period. Oxytocin mean values might to a greater extent reflect characteristics of maternal endogenous oxytocin levels.

#### Influence of Medical Interventions in Connection With Birth

Maternal mean oxytocin levels were significantly lower in mothers who had received both EDA and oxytocin iv, when compared to the effect observed in response to either of the treatments alone. In addition, it was found that oxytocin levels in the control group and in the group who had received oxytocin im were significantly higher than in the group of mothers who had received epidural and oxytocin.

These data support but extend results from a previous analysis of the same data set, in which a significant difference regarding maternal mean oxytocin levels was demonstrated between the group of mothers who had received EDA and those who had received EDA and oxytocin iv (Jonas et al., 2009).

The finding of lower oxytocin levels in response to breastfeeding in mothers who had received EDA *with* oxytocin may seem difficult to explain. However, there is reason to believe that the combination of oxytocin and EDA interferes with feedback mechanisms, such as the Fergusson reflex and the sympathetic fibers mediating pain, in connection with birth, thereby establishing the lower oxytocin levels. As this group of mothers also has signs of higher stress levels, e.g., higher levels of ACTH and cortisol, the lower oxytocin levels may be an expression of higher stress levels in these women (Handlin et al., 2009).

#### Link Between Mean Oxytocin Levels and Sucking

The positive association between mean oxytocin levels and the duration of sucking indicates that more oxytocin is released in response to a longer breastfeeding. The association between lower numbers of previous breastfeeding and higher mean oxytocin levels, which was observed in particular in the control group, may at first sight seem contradictory, because the amount of oxytocin released in response to sucking is known to increase over time (Uvnäs-Moberg et al., 1990). However, basal oxytocin levels are higher the first days after birth because of the high estrogen levels during pregnancy and birth (Uvnäs-Moberg et al., 1990). These elevated levels subside over the first few days. This estrogen-linked elevation of oxytocin levels may explain the relationship between fewer breastfeeding and higher oxytocin levels the first days after birth.

#### Link Between Oxytocin Variance and Infant Weight Loss Since Birth

Interestingly, loss of infant weight was negatively associated with oxytocin variance, possibly indicating that these children sucked less well and therefore caused less milk ejection and received less milk and, as a consequence, would lose more weight after birth. This relationship between the variance of the maternal oxytocin levels and infant weight loss indicates an effect of the intensity of infant sucking which has not been observed before. This finding receives support from studies performed in pigs, in which the number of sucking piglets was associated with the size of the peak of oxytocin released in response to sucking (Algers et al., 1991). Also, women exposed to breast pumping of both breasts release more oxytocin than those mothers who only use breast pumps on one breast (Uvnas Moberg et al., 2020).

#### Medical Interventions Influence Both Infant Rooting and Maternal Oxytocin Levels

In the present study, interventions during birth influenced both the infant and the mother in connection with breastfeeding 2 days after birth. The rooting behavior of the infant (duration and quality) was negatively influenced by administration of EDA, *with* or *without* concomitant administration of intravenous oxytocin. In addition, maternal oxytocin levels were lower in mothers who had received EDA together with oxytocin during birth.

Oxytocin alone given as an intravenous infusion during labor or as an intramuscular injection postpartum to the mother did not exert a negative influence on duration of rooting or quality of rooting in the infant or on maternal oxytocin levels or variance. In fact, a slight positive effect was observed on duration of rooting in the infants and on maternal oxytocin levels in mothers who had received intravenous infusion of oxytocin during birth.

#### Both Rooting and Sucking Influence Infant Weight Loss

A short duration of rooting and a low variance of oxytocin, presumably a consequence of low infant sucking activity, are linked to a greater infant weight loss 2 days after birth. These data suggest that reduced infant pre-feeding and sucking behaviors result in a lower milk production due to an insufficient stimulation of oxytocin and prolactin release (Jonas et al., 2009).

Taken together, EDA *with* or *without* oxytocin infusion in connection with birth may decrease the release of maternal oxytocin and thereby disturb the milk ejection reflex. In addition, infants’ feeding-associated rooting reflex was decreased 2 days after birth if the mother had received EDA in connection with birth. Therefore, EDA and the combination of EDA together with infusion of oxytocin may influence breastfeeding success and consequently infant’s weight gain.

### Limitations and Strengths

In the Swedish setting, it would have been unethical to randomize women to medical interventions, e.g., administration of EDA, and/or to oxytocin infusion. In the present study, mothers were consecutively recruited, capturing the clinical flow of appearance/administration of the medical interventions. Our sample size is small, but the rigorous selection of participants and the meticulous data collection make this study unique and make a significant contribution to this field of research. Our strict inclusion criteria enabled us to include a homogenous group of healthy primiparous mothers who had not experienced any complications during or after birth and who gave birth in a labor ward admitting only women expecting a normal physiological birth. Furthermore, the research group has a long experience of conducting similar studies, and the experimental design is well validated (Nissen et al., 1996). The same two research midwives performed all the data collection. Also, the state of the infants was well standardized, and all infants were fully breastfed on demand. All breastfeeding experiments were performed at approximately the same time of the day, in connection with the first breastfeeding (06:30–08:00). In addition, the frequent blood sampling, 20 samples collected during the 1-h-long experiment, allowed an accurate estimate of oxytocin levels and patterns. In other studies, most often, only a few samples are retrieved from each participant, making the hormonal data less informative. Experts from several fields composed the multidisciplinary research team, which allows a deeper understanding and evaluation of data. Researchers, who were blinded to the interventions the mothers had received during birth, performed all behavioral assessments with an excellent interrater reliability.

### Conclusion

The present data demonstrate that EDA during birth exerts a negative effect on infant rooting and maternal oxytocin release in connection with breastfeeding. The data show a clear effect of infants’ sucking behavior on maternal oxytocin levels, which has not been demonstrated before. The effect caused by medical interventions, EDA *with* or *without* oxytocin infusion, may influence breastfeeding success and infant weight gain. These data suggest that it is of clinical importance to consider the effects of various medical interventions in regard to breastfeeding success.

## Data Availability Statement

The datasets presented in this article are not readily available because our videofilms cannot be anonymized and thus, we cannot share the data. Requests to access the data should be directed to WJ, wibke.jonas@ki.se.

## Ethics Statement

The local ethics committee at Karolinska Institutet, Stockholm, Sweden, had reviewed and approved the study. All study participants provided written, informed consent.

## Author Contributions

WJ performed the data collection. YT performed the behavior assessments with supervision of A-BR-A, EN, and LL. WJ, EN, and LL performed the data analysis. All authors contributed to the study conception and design, discussed and wrote the manuscript, and read and approved the final manuscript.

## Conflict of Interest

The authors declare that the research was conducted in the absence of any commercial or financial relationships that could be construed as a potential conflict of interest.

## Footnotes

1 https://www.mangold-international.com/en/products/software/behavior-research-with-mangold-interact
